# Stability of MRI radiomic features according to various imaging parameters in fast scanned T2-FLAIR for acute ischemic stroke patients

**DOI:** 10.1038/s41598-021-96621-z

**Published:** 2021-08-25

**Authors:** Leehi Joo, Seung Chai Jung, Hyunna Lee, Seo Young Park, Minjae Kim, Ji Eun Park, Keum Mi Choi

**Affiliations:** 1 Department of Radiology and Research Institute of Radiology, Asan Medical Center, University of Ulsan College of Medicine, 86 Asanbyeongwon-Gil, Songpa-Gu, Seoul, 138-736 Republic of Korea; 2 Bigdata Research Center, Asan Institute for Life Science, Asan Medical Center, 88 Olympic-ro 43-Gil, Songpa-Gu, Seoul, 15505 Republic of Korea; 3 Department of Clinical Epidemiology and Biostatistics, Asan Medical Center, University of Ulsan College of Medicine, 86 Asanbyeongwon-Gil, Songpa-Gu, Seoul, 138-736 Korea

**Keywords:** Neuro-vascular interactions, Imaging techniques

## Abstract

From May 2015 to June 2016, data on 296 patients undergoing 1.5-Tesla MRI for symptoms of acute ischemic stroke were retrospectively collected. Conventional, echo-planar imaging (EPI) and echo train length (ETL)-T2-FLAIR were simultaneously obtained in 118 patients (first group), and conventional, ETL-, and repetition time (TR)-T2-FLAIR were simultaneously obtained in 178 patients (second group). A total of 595 radiomics features were extracted from one region-of-interest (ROI) reflecting the acute and chronic ischemic hyperintensity, and concordance correlation coefficients (CCC) of the radiomics features were calculated between the fast scanned and conventional T2-FLAIR for paired patients (1st group and 2nd group). Stabilities of the radiomics features were compared with the proportions of features with a CCC higher than 0.85, which were considered to be stable in the fast scanned T2-FLAIR. EPI-T2-FLAIR showed higher proportions of stable features than ETL-T2-FLAIR, and TR-T2-FLAIR also showed higher proportions of stable features than ETL-T2-FLAIR, both in acute and chronic ischemic hyperintensities of whole- and intersection masks (p < .002). Radiomics features in fast scanned T2-FLAIR showed variable stabilities according to the sequences compared with conventional T2-FLAIR. Therefore, radiomics features may be used cautiously in applications for feature analysis as their stability and robustness can be variable.

## Introduction

The paradigm is shifting from qualitative visual assessment of medical imaging to quantitative data analysis with the development of high-throughput mining of low- to high dimensional data. Radiomic features are considered to be an important alternative for interpretation and analysis of medical images and to predict lesion characteristics with numerous features, from first-order to high-order features^1–4^.

However, radiomic features can have limitations in their reproducibility or stability. The stability of radiomic features is still challenging with a lack of standardization during image acquisition, reconstruction, segmentation and analyses even though standardized image processing and feature computation have allowed radiomic features to be stable^4^. Among the various types of medical imaging, magnetic resonance imaging (MRI) has a variety of imaging acquisition methods and combinations of complicated parameters even in the same imaging sequences, which makes it difficult to apply radiomic features to MRI.

Fast scanned techniques are essential in the acquisition of MRI because of the major limitation of MRI, the need for a long scan time, particularly in emergency situations such as after a suspected cerebral acute ischemic stroke^5^. Fast scanned images have been realized by using echo-planar imaging (EPI), parallel imaging, echo train length (ETL) and recently introduced advanced techniques such as compressed sensing and simultaneous multi-slice acquisition, and so on^6–13^. The various techniques have resulted in a very complicated combination of imaging parameters, which can hamper the acquisition of stable radiomic features.

T2-Fluid attenuated inversion recovery (FLAIR) is very commonly used and essential sequence for the evaluation of cerebral acute ischemic stroke patients^9–11,14–20^. Therefore, T2-FLAIR is an important candidate for the application of radiomic features. However, there have been attempts to reduce the scan time of T2-FLAIR for a long time, which resulted in various parameters of T2-FLAIR. The EPI, parallel imaging, and ETL have been widely used^6,9–12^. Nevertheless, the stability of the radiomic features have been poorly investigated.

We hypothesized that radiomic features from fast scanned T2-FLAIR show variability relative to conventional T2-FLAIR. Therefore, the aim of our study was to investigate the stability of radiomic features from various fast scanned T2-FLAIR images in patients with acute ischemic stroke, and to compare the agreement of the radiomic features with conventional T2-FLAIR as a reference standard.

## Results

### Stability of radiomic features in fast scanned T2-FLAIR compared with conventional T2-FLAIR

The proportions of stable radiomic features in the first group were 13.8% (82/595) in EPI-T2-FLAIR and 5.5% (33/595) in ETL-T2-FLAIR for acute ischemic hyperintensity and 15.6% (93/595) in EPI-T2-FLAIR and 5.1% (30/595) in ETL-T2-FLAIR for chronic ischemic hyperintensity. Proportions of stable radiomic features in the second group were 16.8% (100/595) in TR-T2-FLAIR and 1.8% (11/595) in ETL-T2-FLAIR for acute ischemic hyperintensity and 13.0% (77/595) in TR-T2-FLAIR and 0.7% (4/595) in ETL-T2-FLAIR for chronic ischemic hyperintensity. EPI-T2-FLAIR and TR-T2-FLAIR showed significantly higher proportions of stable radiomic features than those of ETL-T2-FLAIR (p < 0.001). Proportions of stable radiomic features in the first group were 9.7% (58/595) in the EPI-T2-FLAIR intersection and 3.4% (20/595) in the ETL-T2-FLAIR intersection for acute ischemic hyperintensity and 18.0% (107/595) in the EPI-T2-FLAIR intersection and 11.6% (69/595) in the ETL-T2-FLAIR intersection for chronic ischemic hyperintensity. Proportions of stable radiomic features in the second group were 12.1% (72/595) in the TR-T2-FLAIR intersection and 3.9% (23/595) in the ETL-T2-FLAIR intersection for acute ischemic hyperintensity and 9.6% (57/595) in the TR-T2-FLAIR intersection and 1.2% (7/595) in the ETL-T2-FLAIR intersection for chronic ischemic hyperintensity. EPI-T2-FLAIR and TR-T2-FLAIR showed significantly higher proportions of stable radiomic features than those of ETL-T2-FLAIR (p < 0.002). The detailed results are listed in Table 1.

**Table 1:** Reproducibility of radiomics features in fast scanned T2-FLAIR compared with original T2-FLAIR as a reference standard.

Whole	First group			Second group		
	EPI	ETL	P	TR	ETL	P
**Proportions of stable radiomics features (CCC > 0.85)**						
AIH	13.8% (82/595)	5.5% (33/595)	< .001	16.8% (100/595)	1.8% (11/595)	< .001
CIH	15.6% (93/595)	5.0% (30/595)	< .001	12.9% (77/595)	0.7% (4/595)	< .001
Overall	16.0% (95/595)	8.1% (48/595)	< .001	22.9% (136/595)	2.0% (12/595)	< .001
**Median CCC**						
AIH	0.60 (0.13–0.80)	0.58 (0.23–0.75)	.557	0.66 (0.32–0.81)	0.43 (0.16–0.66)	< .001
CIH	0.37 (0.06–0.76)	0.47 (0.16–0.68)	.011	0.44 (0.23–0.67)	0.30 (0.12–0.45)	< .001
Overall	0.62 (0.14–0.82)	0.57 (0.22–0.76)	.607	0.68 (0.32–0.84)	0.47 (0.18–0.67)	< .001

Intersection	First group			Second group		
	EPI	ETL	P	TR	ETL	P
**Proportions of stable radiomics features (CCC > 0.85)**						
AIH	9.7% (58/595)	3.4% (20/595)	< 0.001	12.1% (72/595)	3.9% (23/595)	< 0.001
CIH	18.0% (107/595)	11.6% (69/595)	0.002	9.6% (57/595)	1.2% (7/595)	< 0.001
Overall	8.7% (52/595)	2.5% (15/595)	< 0.001	13.3% (79/595)	3.4% (20/595)	< 0.001
**Median CCC**						
AIH	0.45 (0.10–0.69)	0. 46 (0.15–0.64)	< 0.001	0.56 (0.27–0.73)	0.52 (0.25–0.69)	< 0.001
CIH	0.41 (0.09–0.75)	0.55 (0.28–0.73)	< 0.001	0.46 (0.24–0.63)	0.32 (0.12–0.48)	< 0.001
Overall	0.49 (0.13–0.75)	0.52 (0.21–0.68)	0.348	0.61 (0.31–0.78)	0.55 (0.23–0.73)	< 0.001

*EPI* echo-planar imaging, *ETL* echo train length, *TR* repetition time, *P* p value, *CCC* concordance correlation coefficients, *AIH* acute ischemic hyperintensity, *CIH* chronic ischemic hyperintensity.

Parentheses indicate numbers of radiomics features in proportions of stable radiomics features or indicate 95% confidence intervals.

### Stable radiomic features in fast scanned T2-FLAIR compared with conventional T2-FLAIR

There were no stable radiomic features across acute and chronic ischemic hyperintensities in the first and second groups. Stable radiomic features across acute and chronic ischemic hyperintensities in the first group were 1.01% (1/99) in gray-level run-length matrix (GLRLM) and 2.22% (1/45) in neighboring gray tone difference matrix (NGTDM). Stable radiomic features across acute and chronic ischemic hyperintensities in the second group were 1.01% (1/99) in GLRLM. Stable radiomic features across acute ischemic hyperintensities in the first group were 2.17% (1/46) in the first order, 2.53% (5/198) in gray-level co-occurrence matrix (GLCM), 4.04% (4/99) in GLRLM, 2.22% (1/45) in local binary pattern (LBP), 1.71% (2/117) in gray-level size zone matrix (GLSZM), and 6.67% (3/45) in NGTDM. Stable radiomic features across chronic ischemic hyperintensities in the first group were 4.44% (2/45) in the second order, 2.02% (4/198) in GLCM, 6.06% (6/99) in GLRLM, 4.27% (5/117) in GLSZM, and 4.44% (2/45) in NGTDM. Stable radiomic features across acute ischemic hyperintensities in the second group were 2.17% (1/46) in the first order, 2.02% (4/198) in GLCM, 2.02% (2/99) in GLRLM, 2.22% (1/45) in LBP, 0.85% (1/117) in GLSZM, and 4.44% (2/45) in NGTDM. Stable radiomic features across chronic ischemic hyperintensities in the second group were 1.01% (1/99) in GLRLM, 2.22% (1/45) in LBP, and 2.22% (1/45) in NGTDM.

**Figure 1:**
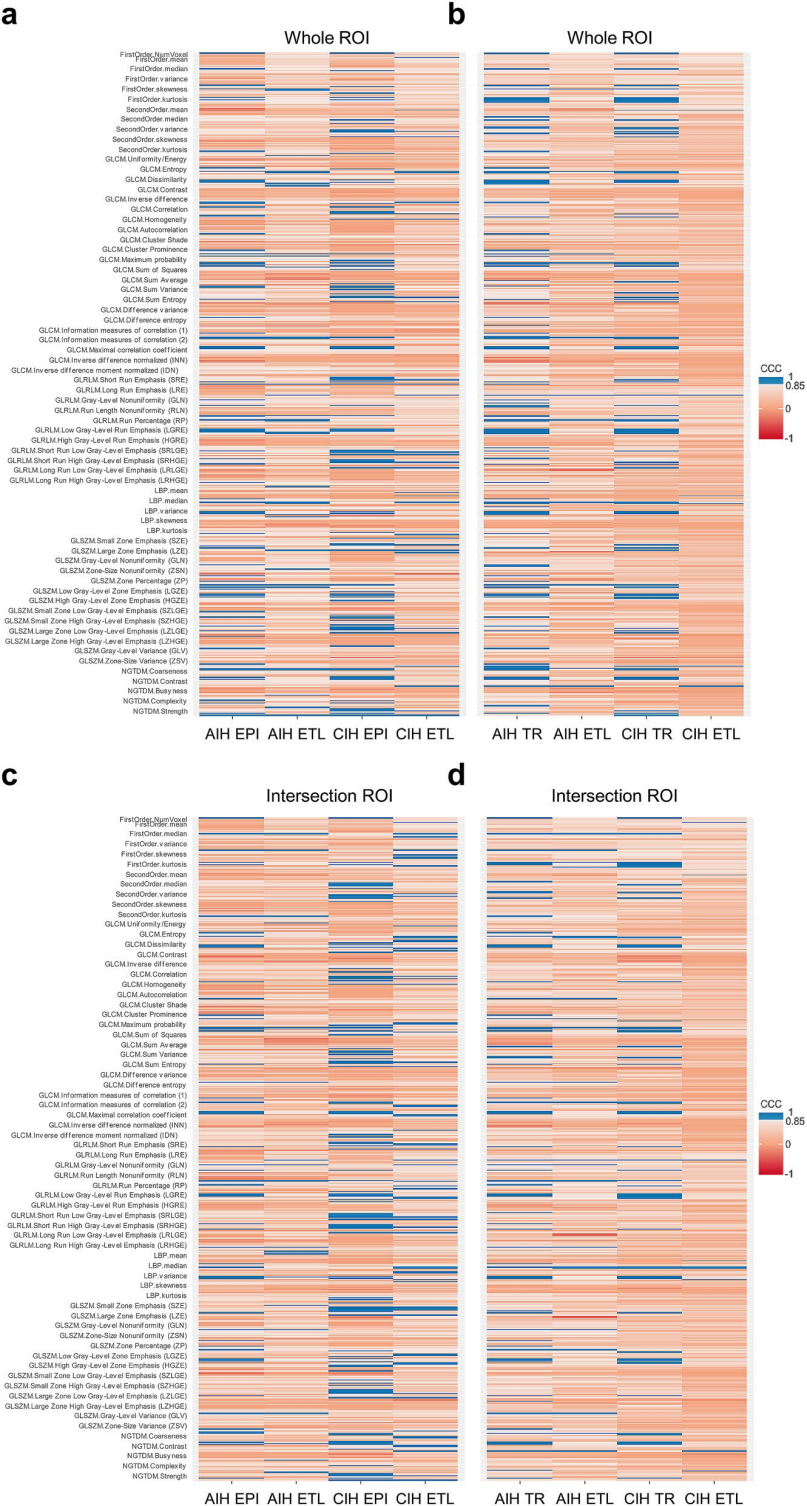
Heat maps of radiomics features extracted from whole- and intersection ROI masks in the first and second groups. Features with CCC > 0.85 were regarded as stable. **(a)** Features from whole ROI masks in the first group. **(b)** Features from whole ROI masks in the second group. **(c)** Features from intersection ROI masks in the first group. **(d)** Features from intersection ROI masks in the second group. *AIH* acute ischemic hyperintensity, *CIH* chronic ischemic hyperintensity, *CCC* concordance correlation coefficients, *ROI* region-of-interest, *EPI* echo-planar imaging, *ETL* echo train length, *TR* repetition time.

**Figure 2:**
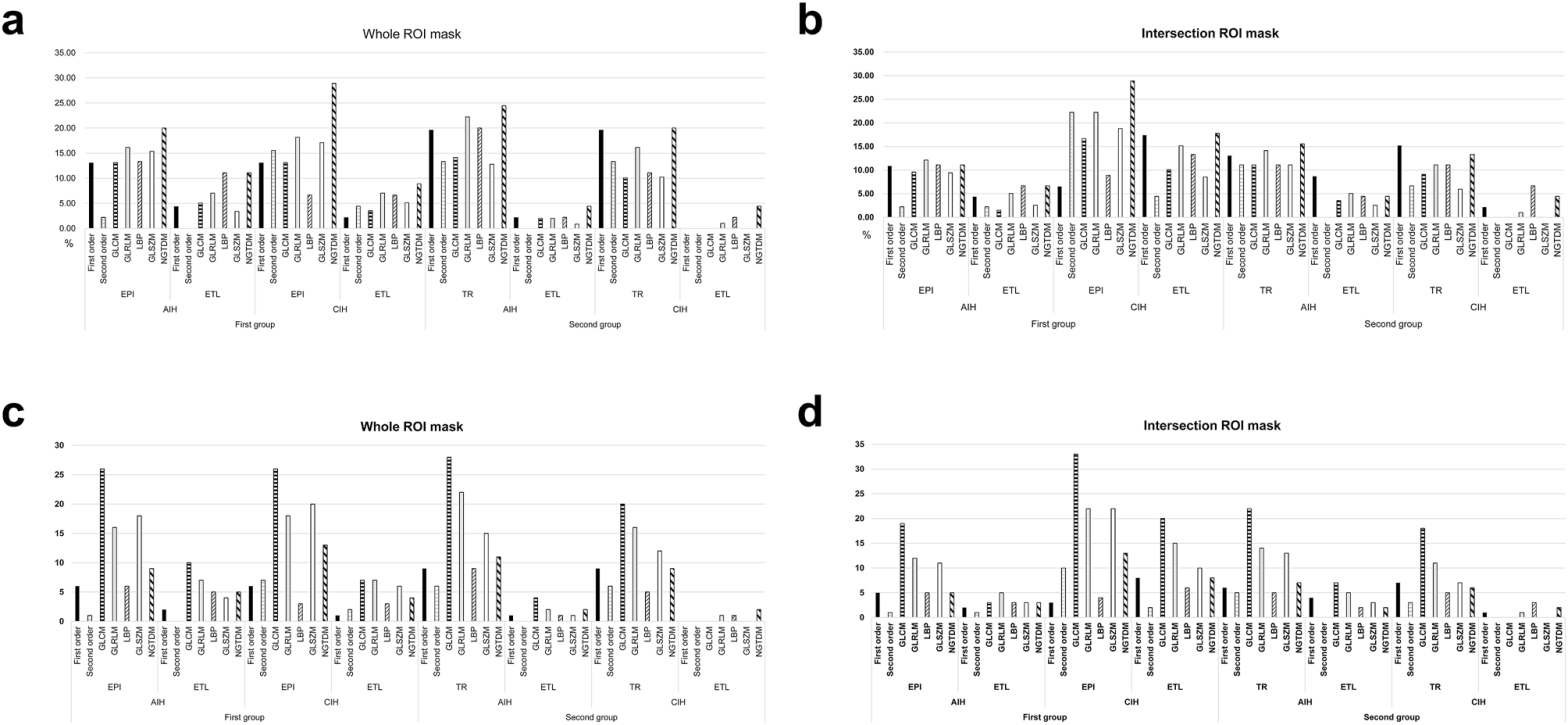
Proportions of stable radiomic features extracted from whole- and intersection ROI masks on each fast scanned T2-FLAIR sequence in the first and second groups. **(a,b)** Proportions of stable radiomic features from whole ROI masks **(a)** and intersection ROI masks **(b)** according to each fast scanned T2-FLAIR. **(c,d)** Numbers of stable radiomic features from whole ROI masks **(c)** and intersection ROI masks **(d)** according to each fast scanned T2-FLAIR. *AIH* acute ischemic hyperintensity, *CIH* chronic ischemic hyperintensity, *ROI* region-of-interest, *EPI* echo-planar imaging, *ETL* echo train length, *TR* repetition time, *GLCM* gray-level co-occurrence matrix, *GLRLM* gray-level run-length matrix, *LBP* local binary pattern, *GLSZM* gray-level size zone matric, *NGTDM* neighboring gray tone difference matrix.

**Figure 3:**
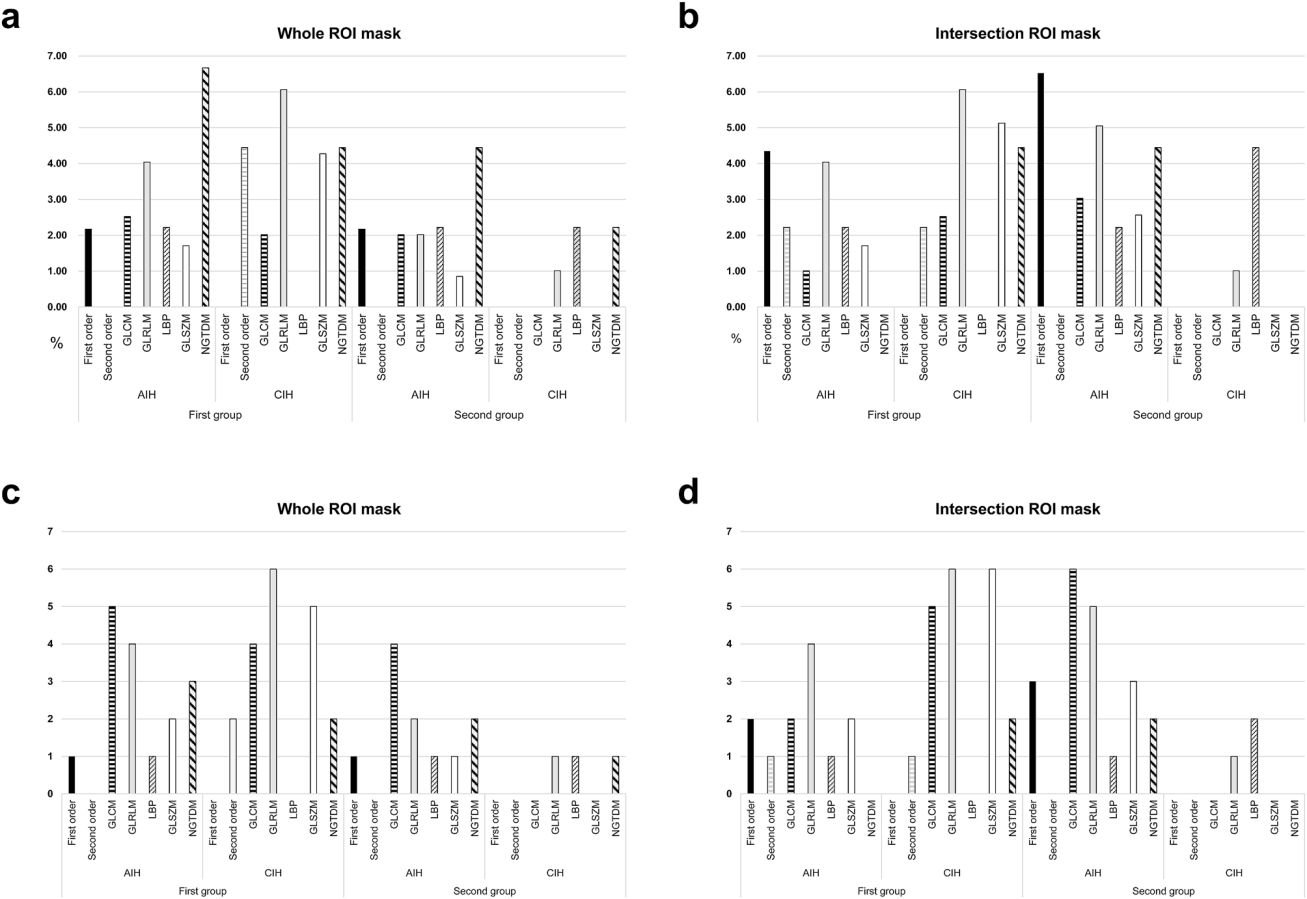
Proportions of stable radiomic features throughout both fast scanned T2-FLAIR sequences extracted from whole- and intersection ROI masks in the first and second groups. **(a,b)** Proportions of stable radiomic features from whole ROI masks **(a)** and intersection ROI masks **(b)**. **(c,d)** Numbers of stable radiomic features from whole ROI masks **(c)** and intersection ROI masks **(d)**. *AIH* acute ischemic hyperintensity, *CIH* chronic ischemic hyperintensity, *ROI* region-of-interest, *EPI* echo-planar imaging, *ETL* echo train length, *TR* repetition time, *GLCM* gray-level co-occurrence matrix, *GLRLM* gray-level run-length matrix, *LBP* local binary pattern, *GLSZM* gray-level size zone matric, *NGTDM* neighboring gray tone difference matrix.

There were no stable radiomic features across acute and chronic ischemic hyperintensities using intersection ROIs in the first group. Stable radiomic features across acute and chronic ischemic hyperintensities in the second group were 1.01% (1/99) in GLRLM and 2.22% (1/45) in LBP. Stable radiomic features across acute ischemic hyperintensities in the first group were 4.35% (2/46) in the first order, 2.22% (1/45) in the second order, 1.01% (2/198) in GLCM, 4.04% (4/99) in GLRLM, 2.22% (1/45) in LBP, and 1.71% (2/117) in GLSZM. Stable radiomic features across chronic ischemic hyperintensities in the first group were 2.22% (1/45) in the second order, 2.53% (5/198) in GLCM, 6.06% (6/99) in GLRLM, 5.13% (6/117) in GLSZM, and 4.44% (2/45) in NGTDM. Stable radiomic features across acute ischemic hyperintensities in the second group were 6.52% (3/46) in the first order, 3.03% (6/198) in GLCM, 5.05% (5/99) in GLRLM, 2.22% (1/45) in LBP, 2.56% (3/117) in GLSZM, and 4.44% (2/45) in NGTDM. Stable radiomic features across chronic ischemic hyperintensities in the second group were 1.01% (1/99) in GLRLM and 4.44% (2/45) in LBP. All of the details mentioned above are shown in Figs. 1, 2 and 3.

## Discussion

This study showed a consistent tendency of higher proportions of reliable features in EPI-T2-FLAIR and TR-T2-FLAIR than ETL-T2-FLAIR in both acute and chronic ischemic hyperintensities and for both whole- and intersection-ROI mask. Therefore, various image acquisitions of T2-FLAIR resulted in unstable radiomic features, which may lead to different radiomic features’ outcomes, such as prediction modeling.

MRI is a useful and sometimes essential imaging modality to identify the infarct core on DWI, and additional useful information can be obtained from various images such as T2-FLAIR or gradient echo (GRE) images, and also allow acquisition of vessel information without the need for contrast media during the evaluation of cerebral acute ischemic stroke patients; however, MRI has a lesser availability and a longer scan time compared to CT^5,21–25^. Therefore, there have been many attempts to reduce the scan time of MRI in cerebral acute ischemic stroke situations, which has resulted in various MRI sequences and parameters being applied in clinical practice^6,9–12^.

Previous studies on fast-scanned T2-FLAIR in acute ischemic stroke showed a consistent superior reliability when compared with that of conventional images^6,9–11^. However, those studies only showed repeatability or reliability in qualitative scoring systems or simple quantitative comparisons, such as signal intensity. In contrast, our study based on radiomic features showed a lower reliability than that of conventional images even though the data was originated from the same registry in the previous study^6^.

The diversity in the image acquisition makes it difficult to apply radiomic features to MRI for cerebral acute ischemic stroke. Imaging acquisition, segmentation, and feature extraction can affect the stability of radiomic features^2^. Ford et al. demonstrated that changes of imaging parameters could lead to variable radiomic features in a phantom study^26^. Minjae et al. only showed that the change in acceleration factors on the same images can affect the stability of radiomic features, and two different under-sampling methods on the same images can show different radiomic features even under the same acceleration factors^27^. Therefore, different imaging parameters even on the same FLAIR sequences may reduce the stability of radiomic features, as in this study. In addition, these obstacles may affect a few published studies on the predictive models developed using MRI radiomic features in cerebral acute ischemic stroke^28–30^. The results from this study showed some stable radiomic features across variable acquisition of T2-FLAIRs and acute and chronic ischemic hyperintensities, but a substantial proportion showed variability. To our knowledge, there is no previous report on the stability of MRI radiomic features according to various imaging parameters in cerebral acute ischemic stroke. The segmentation can affect the stability of radiomic features. Manual segmentation, compared with semi- or automated segmentations, may lead to lower reproducibility in radiomic features^2^. However, many previous studies on radiomic features or high dimensional quantitative analyses using artificial intelligence relied on manual segmentations. In addition, Haarburger et al. showed poor reproducibility of some radiomic features even under automated segmentation methods^31^. The segmentation reproducibility can be influenced by the anatomic location and lesion types^27,32^. In our study, there were some different results between whole and intersection ROI masks, which may be also owing to different sizes of ROI masks and thus different numbers of pixels. Feature extraction can also affect the stability of radiomic features. Li et al. demonstrated the poor stability of radiomic features (no features > 0.85 in concordance correlation coefficient [CCC]) across different extraction combinations^33^.

Studies evaluating stable radiomic features in cerebral acute or chronic ischemic lesions based on multiparameteric variances appear to be lacking, and the reproducibility of radiomic features in brain tumors, including glioblastoma, has been reported and several features belonging to GLRLM were identified as reproducible features^33,34^. GLRLM was also the most reproducible feature in cine balanced steady-state free procession and first-order, and GLCM had the most reproducible features on both T1 and T2 maps in the myocardial radiomic features^35^. However, a phantom study for test–retest reproducibility reported that GLRLM was neither the most robust nor least robust feature class, while GLCM was one of the least robust feature classes across MRI sequences: FLAIR, T1-weighted, and T2-weighted imaging^36^. Our study also showed that GLCM and GLRLM are common stable features in the numbers but some variability was seen in the proportions of stable radiomic features.

This study has several limitations. First, this study was designed as a retrospective study with a small population in a single center. A study population cannot be free from selection bias, which may have affected the deviations in sex and age, and a specific MR machine was adopted. Therefore, further multi-center studies with a larger sample size are necessary. Second, we did not compare all of the T2-FLAIRs simultaneously because it is hard to obtain all of the T2-FLAIRs at the same time in a cerebral acute ischemic stroke situation. Third, this study presented only the stabilities of radiomic features in T2-FLAIRs of acute ischemic stroke and an evaluation of the stability of identification or prediction models influencing the treatment options for stroke outcomes using radiomic features from variable T2-FLAIRs is necessary.

In conclusion, the fast-scanned T2-FLAIR showed small proportions of stable radiomic features and variable stability of radiomic features among those in terms of the agreements with conventional T2-FLAIR. Therefore, even if the images in the same sequence have different parameters, the radiomic features obtained from the images may be used with caution.

## Methods

### Study population

From May 2015 to June 2016, data on 296 patients undergoing MRI at a single tertiary hospital for symptoms of acute ischemic stroke were retrospectively collected. Among them, 118 patients underwent echo-planar imaging (EPI)-T2-FLAIR and echo train length (ETL)-T2-FLAIR and 178 patients underwent ETL-T2-FLAIR and repetition time (TR)-T2-FLAIR simultaneously. In total, 79 patients showed acute ischemic hyperintensity and 89 patients showed chronic ischemic hyperintensity on simultaneous acquisition of EPI- and ETL-T2-FLAIR, who were classified to the first group, and 112 patients showed acute ischemic hyperintensity and 127 patients showed chronic ischemic hyperintensity on simultaneous acquisition of ETL- and TR-T2-FLAIR, who were classified to the second group for comparable paired data analysis. The detailed demographics of the patients are presented in Table 2. The data on patients were collected from the fast stroke MRI registry in our institute^6^. The institutional review board of Asan Medical Center approved the present study, and the requirement for informed consent was waived. The data was analyzed in compliance with the International Council for Harmonization of Technical Requirements for Registration of Pharmaceutical for Human Use: Guideline for Good Clinical Practice (ICH GCP).

**Table 2:** Demographics of the patients.

	Acute ischemic lesion		P value	Chronic ischemic lesion		P value
	EPI-ETL (n = 79)	ETL-TR (n = 112)		EPI-ETL (n = 89)	ETL-TR (n = 127)	
Age (years)^a^	67.9 ± 13.2 (38–92)	67.8 ± 13.4 (22–95)	0.95	69.4 ± 10.9 (38–92)	72.4 ± 9.4 (38–95)	0.01
Sex (M:F)	46:33	73:39	0.41	52:37	74:53	0.91
Body mass index (kg/m^2^)^a^	23.9 ± 3.5 (16.4–32.1)	24.1 ± 3.4 (13.8–36.6)	0.76	23.3 ± 2.9 (15.1–32.1)	23.4 ± 3.4 (13.8–36.6)	0.86
Hypertension^b^	54 (68.4)	70 (63.1)	0.5	64 (71.9)	92 (73.0)	0.95
DM^b^	28 (35.4)	29 (26.1)	0.21	33 (37.1)	43 (34.1)	0.73
Hyperlipidemia^b^	34 (43.0)	37 (33.6)	0.21	35 (39.3)	43 (34.7)	0.5
Smoking history^b^	32 (40.5)	43 (38.7)	0.89	36 (40.4)	84 (33.3)	< 0.01
History of stroke^b^	17 (21.5)	24 (21.6)	< 0.01	25 (28.1)	46 (37.1)	0.27
Family history of stroke^b^	19 (24.1)	33 (29.7)	0.51	20 (22.5)	30 (24)	0.97
History of coronary disease^b^	26 (32.9)	42 (37.8)	0.62	30 (33.7)	45 (36)	0.91
Onset to imaging time	258 (113–683)^c^	223 (123 –700)^c^	0.65	261 (123.5–576)^c^	172 (117.5–306.5)^c^	0.10

^a^Numbers indicate mean ± standard deviation and the parentheses indicate the range.

^b^Parentheses indicate the range.

^c^Median (nterquartile range).

### Image acquisition

**Figure 4:**
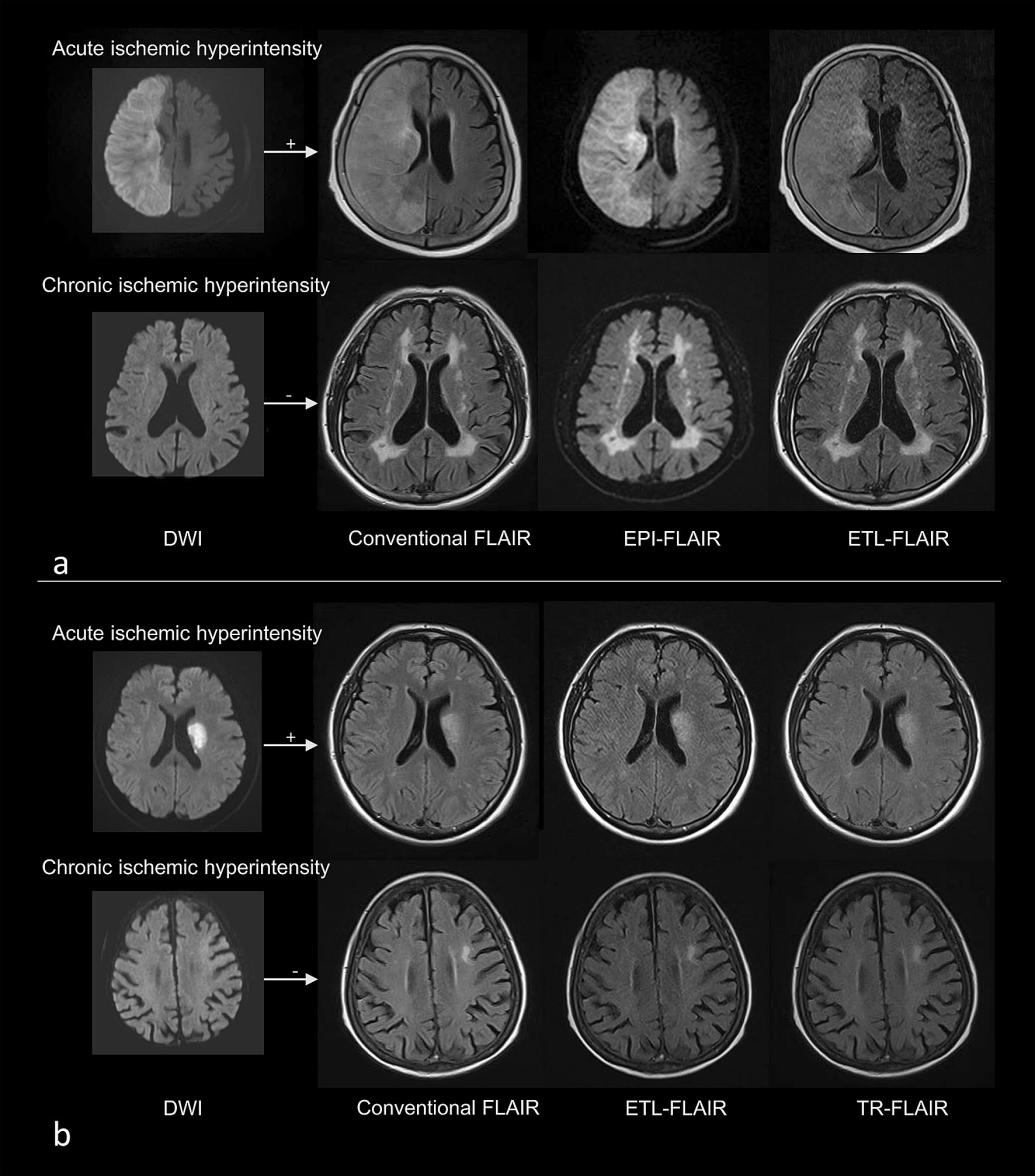
Acute and chronic ischemic hyperintensities on conventional T2-FLAIR and fast scanned T2-FLAIR with respect to DWI. **(a)** First group with conventional T2-FLAIR, EPI-T2-FLAIR, and ETL-T2-FLAIR. **(b)** Second group with conventional T2-FLAIR, ETL-T2-FLAIR and TR-T2-FLAIR. *DWI* diffusion-weighted image, *FLAIR* fluid attenuated inversion recovery, *EPI* echo-planar imaging, *ETL* echo train length, *TR* repetition time.

All T2-FLAIR was scanned on a 1.5-T scanner (Magnetom Avanto; Siemens Healthineers). The scan times were 128 s for conventional T2-FLAIR, 45 s for EPI-T2-FLAIR, 74 s for ETL-T2-FLAIR, and 79 s for TR-T2-FLAIR. The detailed scan parameters for the conventional and fast T2-FLAIR were as previously reported^6^ and are listed in Table 3 and representative images are presented in Fig. 4.

**Table 3:** T2-FLAIR protocols.

	T2-FLAIR			
	Conventional	EPI	ETL	TR
TR/TE	9000/109	9000/101	9000/102	5560/109
Inversion time	2500	2000	2500	1930
Flip angle	150	180	150	150
Matrix	256 × 218	128 × 128	192 × 192	256 × 256
Field of view	210 × 184	230 × 230	210 × 184	210 × 210
Number of slices	20	20	20	20
Slice thickness (mm)	5	5	5	5
Slice gap (mm)	2	2	2	2
ETL	21	128 (EPI)	32	21
Acceleration factor	2	2	2	2
NEX	1	2	1	1
Scan time	128	45	74	79

*EPI* echo-planar imaging, *ETL* echo train length, *TR* repetition time, *TE* echo time, *NEX* number of excitations.

### Image analysis

**Figure 5:**
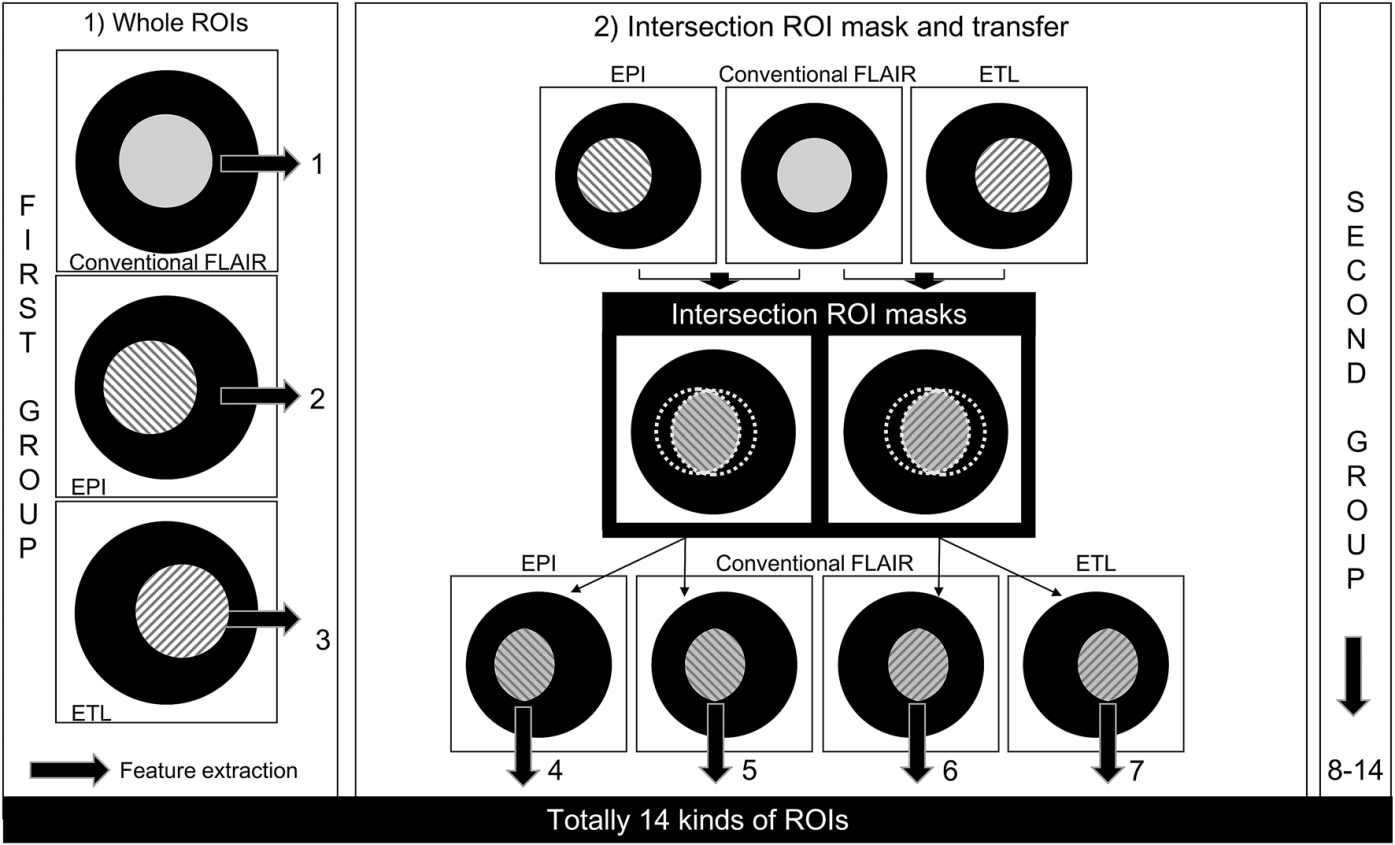
The outline of the image analysis focused on the types of ROI masks used in this study. Feature extraction was performed from each conventional and fast scanned T2-FLAIR using both whole-(× 3) and intersection ROI masks (× 4) for both the first and second groups (× 2). *FLAIR* fluid attenuated inversion recovery, *ROI* region-of-interest, *EPI* echo-planar imaging, *ETL* echo train length, *TR* repetition time.

The segmentations of acute- and chronic ischemic hyperintensities were conducted as described in a previous report^6^. We defined acute ischemic hyperintensity as a T2-FLAIR high signal intensity within acute infarcts on diffusion weighted images (DWI)^15,17^, and chronic ischemic hyperintensity as a hyperintensity outside of acute infarcts on DWI. The segmentation of region-of-interest (ROI) mask was done by one research assistant (K.M.C. with 5 years of experience in stroke imaging) using an in-house program for semiautomatic segmentation (based on ImageJ software; National Institutes of Health, Bethesda, MD). The intersection ROI mask between the respective ROI mask of the conventional and fast T2-FLAIR was obtained after the coregistration and then each intersection ROI mask was transferred into the respective conventional and fast T2-FLAIR images. The intersection ROI mask was used to compare the radiomic features from different T2-FLAIRs without ROI mask differences. A total of 14 kinds of ROI masks were obtained as follows: 3 ROIs from conventional-, EPI-, and ETL-T2-FLAIR in the first group; 2 intersection ROIs from conventional and EPI-T2-FLAIR, and 2 intersection ROIs from conventional and ETL-T2-FLAIR in the first group; 3 ROIs from conventional-, ETL-, and TR-T2-FLAIR in the second group; 2 intersection ROIs from conventional and ETL-T2-FLAIR, and 2 intersection ROIs from conventional and TR-T2-FLAIR in the second group (Fig. 5). From the ROIs, 595 radiomics features were extracted and concordance correlation coefficients (CCC) for radiomic features were calculated between fast scanned and conventional T2-FLAIR in each group.

### Radiomic features

Radiomic features were extracted with Matlab R2016a (The Mathworks, Natick, MA): first-order features, texture features, and wavelet-transformed features^37,38^. The first-order features were acquired based on the histogram analyses of pixel values within the region-of-interest. The second-order features or texture features were as follows: gray-level co-occurrence matrix (GLCM), gray-level run-length matrix (GLRLM), local binary pattern (LBP), gray-level size zone matric (GLSZM), and neighboring gray tone difference matrix (NGTDM). The wavelet transformations extracted additional features. The radiomic features extraction was done under the Imaging Biomarker Standardization Initiative^39^. Finally, 595 radiomic features were extracted with 46 first-order features, 61 texture features (5 s order, 22 GLCM, 11 GLRLM, 5 LBP, 13 GLSZM, 5 NGTDM) and 488 wavelet features (× 8; 40 s order, 176 GLCM, 88 GLRLM, 40 LBP, 104 GLSZM, 40 NGTDM).

### Statistical analysis

The stability of the radiomic features was evaluated using CCC between the features extracted from the conventional- and fast scanned T2-FLAIR based on Lin’s definition^40^. The proportions of stable radiomic features were compared between EPI and ETL in the first group and between TR and ETL in the second group using McNemar’s test. Radiomic features with a CCC of higher than 0.85 were considered to be stable. All statistical analyses were performed using the MedCalc 15.6.1 software package (MedCalc Software) and R version R 3.3.3 (R Foundation for Statistical Computing; http://www.R-project.org, 2016).

## Data Availability

The datasets collected during and/or analyzed during the current study may be available from the corresponding author on reasonable request and in compliance with ethical standards under an approval of the local institutional review board.
